# Emotional Granularity and Cognitive Reappraisal Affect Social Anxiety and Interpersonal Relationships in Adolescents: A Bayesian Network Analysis

**DOI:** 10.1155/da/8658973

**Published:** 2025-02-27

**Authors:** Wen Lian, Xinyi Zhu, Tingting Xu, Lu Fan, Yuqi Sun

**Affiliations:** ^1^Department of Psychology, School of Education, Wenzhou University, Wenzhou 325035, China; ^2^Department of Psychology, Jing Hengyi School of Education, Hangzhou Normal University, Hangzhou 311121, China; ^3^School of Mental Health, Wenzhou Medical University, Wenzhou 325035, China; ^4^Zhejiang Provincial Clinical Research Center for Mental Disorders, The Affiliated Wenzhou Kangning Hospital, Wenzhou 325035, China

**Keywords:** adolescent, emotional granularity, emotional regulation, interpersonal relationship, network analysis, social anxiety

## Abstract

**Background:** Emotional granularity (EG), the ability to finely distinguish emotional experiences, plays a crucial role in emotion regulation and social interactions. This study measures EG using a standardized experimental procedure and assesses related variables through questionnaires. We employ both undirected graphical Gaussian models (GGM) and directed Bayesian network analysis (NA) to investigate how positive EG (PEG) and negative EG (NEG), in conjunction with emotion regulation, uniquely influences social anxiety and interpersonal relationships.

**Methods:** The sample comprised 407 junior high school students from China, aged 13–14 years old. We utilized the Photo Emotion Differentiation Task (PED task), Emotion Regulation Scale (ERS), Interpersonal Relationship Comprehensive Diagnostic Scale (IRCDS), and Adolescent Social Anxiety Scale (SAS-A) to measure emotional differentiation, emotion regulation, interpersonal relationships, and social anxiety, respectively.

**Results:** The undirected network highlights a pronounced relationship between interpersonal relationships and social anxiety. “Socialization and Friendship Distress” shows the highest expected influence, followed by “Social Avoidance and Distress in General Situations”, and “Conversational Distress”. Bayesian NA indicates that both PEG and NEG, along with cognitive reappraisal (CR), influence social anxiety and interpersonal relationships. PEG directly affects social anxiety features, whereas NEG is influenced by them. Additionally, females experience more severe issues with interpersonal relationships and social anxiety than males.

**Conclusion:** The study revealed that individuals with lower positive EG directly impact social anxiety and interpersonal issues, and indirectly affect CR. Conversely, negative EG is mainly influenced by social anxiety symptoms and directly affects CR strategies. These findings highlight the importance of EG in adolescent mental health, suggesting that enhancing emotional differentiation could effectively address social anxiety and foster healthier interpersonal relationships.

## 1. Introduction

In contemporary society, individuals face substantial daily stress, resulting in an increasing number of people, particularly adolescents, seeking emotional support and value. The inability to differentiate emotions can lead to various interpersonal and social issues, highlighting the importance of effectively managing these emotions. Emotional experiences are integral to daily life and encompass multiple states, but individuals may struggle when emotions are less distinct [1]. In such cases, accurately identifying and defining these emotions can become relatively complex. Barrett first introduced the concept of emotional granularity (EG), suggesting that it represents the ability of individuals to finely differentiate their emotional experiences [1, 2]. In subsequent studies, researchers have gradually enriched EG, which is also known as “emotion differentiation”, and refer to the specificity of emotional representations/experiences, specifically individual differences in an individual's ability to make fine-grained, subtle distinctions between similar emotional states [3, 4].

Based on valence, EG is divided into positive EG (PEG) and negative EG (NEG). Until now, research on NEG outweighs that on PEG. However, numerous studies have confirmed the benefits of PEG, with some research indicating that individuals with higher PEG report better stress-coping abilities [5]. In contrast, the majority of studies on NEG focus on mental health [6], psychological disorders [7], and maladaptive behaviors [8]. As research on EG has expanded, many researchers have begun to make more precise measurements and have attempted to utilize standardized laboratory procedures. Measuring EG typically involves having participants report their emotional experiences multiple times across various situations, allowing for a detailed assessment of their ability to differentiate between subtle emotional states. Common methods for measuring EG include experiential sampling (ES) and Ecological Momentary Assessment (EMA) [9], as well as standardized laboratory tasks [4]. The primary distinction is that, in laboratory settings, emotions are artificially induced rather than naturally experienced in daily life. While this approach may lack ecological validity, it offers a more straightforward and controlled operational method for assessing EG. Studies indicate that EG follows a U-shaped developmental trajectory: it declines from childhood to adolescence and increases from adolescence to adulthood [10]. Adolescence would be a period of low emotion granularity in which emotions co-occur more frequently [11, 12]. This study focuses on junior high school students aged 13–14, who generally have weaker emotion recognition and regulation abilities due to limited emotional knowledge. Research suggests that as emotional knowledge increases, individuals' ability to differentiate emotions improves. Increasing emotional knowledge through interventions can significantly improve emotional differentiation, particularly in managing negative emotions [13].

EG plays a crucial role in emotion regulation. Individuals with a high ability to accurately recognize and differentiate their negative emotions are typically more motivated to actively manage their emotions [1]. This is because the ability to clearly distinguish between feelings such as anger, sadness, or fear, enhances the likelihood of adopting effective emotion regulation strategies [1, 5]. Dodge and Gross consider emotion regulation as the process by which individuals consciously and unconsciously modulate their emotional experiences and expressions [14, 15]. This process encompasses five distinct strategies: situation selection, situation modification, attentional deployment, cognitive change, and response modulation [16]. These strategies form the basis of five families of emotion regulation techniques. Research indicates that EG can serve as a protective factor under significant stress or during intense negative emotional experiences. It enables individuals to better cope with challenges and situations by providing a nuanced understanding of their emotional states [17]. Some studies have indicated that low EG is not directly associated with the selection of emotion regulation strategies but rather with their effectiveness, showing a stronger correlation with the intensification of negative emotions [18]. Additionally, the study has shown that individuals with higher EG tend to have relatively higher working memory capacity [19]. Numerous studies have confirmed that emotion regulation is related to working memory capacity, likely due to the reduced neural resources required during successful emotion regulation [20, 21]. In adolescents, reduced working memory ability is associated with poor emotion regulation [22]. Therefore, it is evident that the relationship between EG and emotion regulation is closely intertwined with internal cognitive mechanisms.

The ability to appropriately express and control emotional experiences is crucial during the three stages of establishing interpersonal relationships [23]. The concept of “interpersonal relationships” was first introduced by the American Personnel Management Association in the early 20th century [24]. Interpersonal relationships can be categorized into two types: broad and narrow. Interpersonal relationships refer to the emotional connections formed between people through direct interactions [25]. Zheng [26] defines interpersonal relationship difficulties as various problems arising in the areas of making friends, interpersonal communication, social interactions, and relationships with the opposite sex. For middle school students, interpersonal relationships include peer relationships (both same-sex and opposite-sex peers), teacher–student relationships, parent–child relationships, and relationships with unfamiliar adults [27]. How individuals regulate their emotions significantly impacts their relationships and overall well-being [14].

A study involving college students found that those with higher scores in emotional regulation had more positive relationships, experienced fewer conflicts with close friends, and received greater emotional support from their parents [28]. Gross and John [29] discovered that individuals who favor cognitive reappraisal (CR) strategies are more likely to be accepted by others, maintain more harmonious interpersonal relationships, and enjoy more intimate relationships, Conversely, those who prefer expressive suppression (ES) strategies tend to experience less favorable outcomes in their relationships. Further research has revealed that while ES strategies can reduce the intensity of negative emotional experiences, they do not fundamentally alter their nature. Prolonged suppression of negative emotions can lead to their accumulation, intensification, and adverse effects on interpersonal relationships, exacerbating interpersonal distress [30]. Therefore, neither regulation strategy is inherently superior or inferior; each has its appropriate context for application.

Research indicates that individuals with lower EG are more likely to exhibit social avoidance behavior during rumination, which is a typical characteristic of social anxiety [31]. Social anxiety is one of the most prevalent mental health disorders, characterized by a significant fear of and avoidance of social or performance situations [32]. The prevalence of social anxiety disorder (SAD) among adolescents varies widely, ranging from approximately 0.8% to 22.9% [33]. This variation is influenced by factors including geography, culture, methodology, and gender. Additionally, social anxiety significantly impacts social relationships, and this effect can be exacerbated by dysfunctional family dynamics and poor general relationships [34].

Clinical studies indicate that patients with SAD exhibit reduced NEG compared to healthy controls, though the clarity of PEG in these patients has not been established [35]. Individuals with SAD are more likely to employ ES as a strategy for emotion regulation, rather than CR [36]. Furthermore, studies have found that the effectiveness of emotion regulation strategies inversely correlates with the severity of social anxiety symptoms in both adolescents and children [37, 38]. This suggests that as social anxiety increases, the efficacy of employed emotional regulation strategies decreases. Adolescents with higher levels of social anxiety often struggle with psychosocial adaptation and are more susceptible to peer relationship issues [39]. These adolescents are also more likely to employ ES strategies, which are less effective as emotional regulation tactics. A study conducted on college freshmen reached a consistent conclusion [40]. Studies also show that low peer acceptance correlates significantly with social anxiety in both genders. Among girls, specific risk factors include a lack of close friendships, negative friendship experiences, and relational victimization [41].

In recent years, network research has garnered significant attention within the field of psychological sciences. Network analysis (NA) has emerged as a powerful tool for understanding mental disorders, conceptualizing them not as isolated entities but as complex networks of interacting symptoms. Grounded in graph theory, NA provides graphical visualization of these networks, revealing causal relationships between variables. Two key methods in psychological NA are Graphical Gaussian Model (GGM) and Bayesian networks. GGM networks, with their partial correlations, help identify core symptoms, uncover correlations across disorders and pinpoint influential nodes. A Bayesian network is a graphical model where nodes represent variables, and arrows indicate probabilistic dependencies between them [42]. They highlight “central symptoms” that are pivotal in network structure and “bridge symptoms” that link different disorders, which are crucial for understanding comorbidity. Bayesian networks, characterized by directed acyclic graphs (DAGs) and conditional probabilities, reveal causal influences between symptoms and identify upstream nodes as primary intervention targets. NA, by identifying central and bridge symptoms, elucidates crucial mechanisms in psychological disorders, thereby guiding effective treatments. The integration of NA enhances understanding of psychopathology and informs targeted interventions to improve mental health. This methodological approach is widely applied in psychology, uncovering intricate interdependencies and providing deeper insights into human behavior and mental processes.

In this research, we utilized standardized experimental paradigms to examine EG, addressing not only the frequently studied negative NEG but also the critical role of PEG in adolescent mental health and social interactions. While existing studies have enriched our understanding of EG, our research seeks to further investigate the intricate relationships among the variables using novel methodological approaches. Considering the influence of EG on emotional regulation, our study employs GGM NA and Bayesian networks to explore both undirected and directed relationships among PEG and NEG, emotional regulation, interpersonal relationships, and social anxiety [1]. This investigation focuses on these four variables, constructing a domain-level network and developing Bayesian networks with PEG and NEG as pivotal elements. The aim is to identify core psychological traits and causal relationships among these variables, thereby enhancing our comprehension of their dynamics.

## 2. Materials and Methods

### 2.1. Participants

This cross-sectional study was conducted in collaboration with a school psychologist to facilitate the investigation. Utilizing a cluster sampling method, we recruited 449 students from a secondary school in Zhejiang Province, China. Cases with negative intraclass correlation coefficient (ICC) values, which measure rating consistency across contexts and image presentations, were excluded from the analysis [43]. ICC values, ranging from 0 to 1, are interpretable, with higher ICC values indicating lower levels of EG, consistent with prior studies [5, 44, 45]. After excluding these cases and those with missing data for certain variables, we obtained 407 valid questionnaires. The final sample consisted of 204 male and 203 female middle school students, with a mean age of 13–14 years. This research, including the consent procedure, was approved by the Ethics Committee of Wenzhou University.

### 2.2. Measures

#### 2.2.1. Emotional Granularity

**Figure 1:**
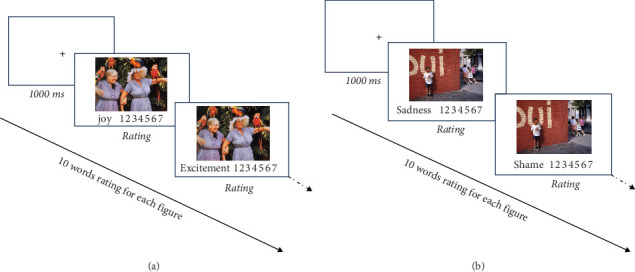
Schematic of a trial of the photo emotion differentiation task (PED task) for positive emotional granularity (A) and negative emotional granularity (B).

The Emotion Differentiation task, developed by Erbas and henceforth referred to as the Photo Emotion Differentiation task (PED-task) [46], provides a convenient method for measuring EG. Participants were asked to evaluate their emotional responses using standardized emotional figures from the International Affective Picture System [47]. Based on the literature, 10 emotion words were selected, and 3–5 representative figures for each emotion were initially chosen from the IAPS. Thirty participants were randomly selected for a pilot study to assess these figures on a Likert 7-point scale for valence, arousal, dominance, and the emotion most closely associated with each figure. Following this evaluation, images were finalized for each emotion word. The final selection comprised 10 positive emotional figures (relaxation, gratitude, awe, satisfaction, challenge, hope, excitement, happiness, joy, and pride) and 10 negative emotional figures (fear, anxiety, anger, disgust, depression, sadness, loneliness, shame, frustration, and guilt). In the formal experiment, participants were randomly presented with an emotional figure, followed by a list of emotional words. They rated the intensity of each emotion experienced while viewing the image. The schematic of a trial of the Photo Emotion Differentiation task (PED task) is shown in Figure 1. Each participant rated 10 words for each figure, and there are 10 figures for both PEG and NEG. The PED task for both PEG and NEG was completed in the school's computer lab, while other questionnaire tasks were conducted in the classroom using paper-based tests.

#### 2.2.2. Emotion Regulation Scale

The Emotion Regulation Scale (ERS) is an instrument developed based on Gross's Emotion Regulation Questionnaire [29]. The Chinese version of the scale was revised by Wang et al. to align with the emotion regulation process mode [48]. The ERS comprises 14 items, divided into two dimensions: ES and CR. Participants rate each item on a 7-point scale, ranging from 1 (“completely disagree”) to 7 (“completely agree”). Scores for each dimension are calculated by summing the scores of the corresponding items, with higher scores indicating more frequent use of the respective emotion regulation strategy. The internal consistency of the ERS in this sample of middle school students was measured by Cronbach's alpha, yielding a value of 0.771.

#### 2.2.3. The Interpersonal Relationship Comprehensive Diagnostic Scale

The Interpersonal Relationship Comprehensive Diagnostic Scale (IRCDS), developed by [26], consists of 28 items categorized into four dimensions: socialization and friendship, heterosexual interaction, interpersonal conversation, and interpersonal interaction. Each dimension comprises seven items, and participants respond with either “yes” or “no”. Higher scores indicate greater levels of distress in interpersonal relationships. Specifically, scores exceeding 8 suggest a certain degree of interpersonal distress while scores surpassing 15 indicate severe distress. In this study, the scale demonstrated good internal consistency, with a Cronbach's alpha coefficient of 0.852.

#### 2.2.4. Social Anxiety Scale for Adolescents

The simplified version of the Social Anxiety Scale for Adolescents (SAS-A) is adapted from a shortened version developed by Benner and Graham [49]. The SAS-A consists of 12 items across three dimensions: Fear of Negative Evaluation (FNE), Social Avoidance and Distress in New Situations (SAD-New), and Social Avoidance and Distress in General Situations (SAD-G). Participants rate each item on a 5-point Likert scale ranging from 1 (completely disagree) to 5 (completely agree). The scale demonstrates good reliability and validity, making it suitable for assessing social anxiety among junior middle school students. In this study, the SAS-A showed excellent internal consistency, with a Cronbach's alpha coefficient of 0.902.

The specific items of the scales are all included in the Table S4.

### 2.3. Data Analysis

#### 2.3.1. Descriptive Statistics

JASP software (Jeffrey's Amazing Statistics Program; version 0.18.3) was utilized for statistical analyses. This included computing descriptive statistics, conducting independent sample *t*-tests, performing correlation analysis, calculating effect size (Cohen's d), and determining Cronbach's *α* coefficient.

#### 2.3.2. Network Estimation

The NA was conducted using the R programming language, version 4.3.3 [50]. Gaussian graphical models (GGM) were employed to construct the network and the qgraph package (version 1.9.8) of R software was used for network visualization [51, 52]. In GGM, nodes represent variables, and edges represent partial correlation coefficients between variables [53]. The color and thickness of edges denote both the polarity and magnitude of correlations, with red hues indicating negative correlations and green hues indicating positive correlations. Thicker edges correspond to stronger correlations, while the absence of an edge between two nodes signifies conditional independence. Given that the complexity of GGM increases quadratically with the number of nodes, leading to many spurious edges. We employed the Least Absolute Shrinkage and Selection Operator (LASSO) to maintain sparsity by excluding spurious edges and effectively regularizing the GGM [54, 55]. LASSO uses a tuning parameter to control regularization and improve model selection. In qgraph, the default settings incorporate the Extended Bayesian Information Criterion (EBIC) with LASSO, known for its consistent model selection performance, especially with limited samples and increasing parameters [56].

Additionally, we utilized the R package mgm (version 1.2.14) to estimate a network model and calculate the predictability of each node [57]. This involved predicting each node using all other nodes and measuring the proportion of explained variance. A value of 0 indicates no explanation by other nodes, while a value of 1 indicates perfect prediction, representing the percentage of variance explained for each node within the network.

#### 2.3.3. Centrality and Stability

To assess the centrality indices of nodes in the network, including strength centrality, closeness centrality, and betweenness centrality, we utilized the bootnet package in R software (version 1.6) for plotting and JASP (Version 0.18.3) for numerical calculations. These centrality indices are essential for evaluating the significance of individual nodes within the network. Furthermore, the expected influence calculated using qgraph is a key metric for identifying core symptoms. This metric primarily measures the importance of nodes based on their influence and ability to disseminate information within the network [58].

We utilized networktools package (version 1.5.2) to compute the bridge centrality indices of the nodes. Bridge centrality assesses the influence of nodes that connect different communities within a psychological network, emphasizing their role in facilitating crucial interactions. This assessment includes four metrics: strength, closeness, betweenness, and expected influence, higher expected influence values indicate a greater risk of community transition, highlighting nodes that play a significant role in the interconnectedness of the network [59].

We employed the correlation stability coefficient (CS coefficient) to assess the robustness of centrality indices. The CS coefficient indicates the maximum proportion of cases that can be removed while preserving the expected correlation between original and subset-derived centrality indices. CS (cor = 0.7) indicates that up to 95% of cases can be dropped while maintaining a correlation of 0.7 or higher. For meaningful differentiation in centrality, the CS coefficient should not fall below 0.25 and preferably exceed 0.50 [60]. Additionally, we utilized the nonparametric Bootstrap method to evaluate the precision of edge weight estimation in networks. This method constructs confidence intervals for edge weights and validates the reliability and robustness of network models, thereby ensuring accuracy in model selection and parameter estimation.

#### 2.3.4. Bayesian NA

Bayesian networks (BNs), structured as DAGs, consist of nodes and directed edges, representing causal influences between these nodes [61]. Each node in a Bayesian network comes with a conditional probability distribution, facilitating the identification and quantification of causal relationships among the nodes. For example, in such networks, an edge from node A to node B (A→B) signifies that A causally influences B. This structure distinguishes BNs from models like the GGM, as they explicitly represent causal directions.

BN analysis was conducted using the bnlearn package (version 4.9.3) in R employing the Hill Climbing (HC) algorithm to estimate the optimal network structure [42]. By constructing DAGs for the BNs, we identified potential causal relationships between nodes. To ensure the stability of the network structure, we utilized bootstrapping methods, generating BNs from random samples and calculating the frequency of edge occurrences. High-frequency edges were used to create an averaged network. Linear models were fitted for each subnode in the averaged BN using the method of least squares [62]. A separate averaged bootstrapped BN was constructed for PEG and NEG, respectively. In the BNs derived from bootstrap samples, edges with a probability exceeding 85% are depicted, and their directions, with a probability over 50%. Edge thickness was correlated with the magnitude of node influence, indicating a stronger effect with greater thickness. Edges were ranked in descending order of their influence strength.

#### 2.3.5. Network Comparison of Test

To assess the impact of gender on the network structure, a network comparison test was conducted to examine differences between male and female participants. This analysis used the R package Network Comparison Test (version 2.2.2) with 1000 permutations, comparing the network structures and global strengths of the two gender-specific models.

## 3. Results

### 3.1. Descriptive Statistics

Table 1 presents the results of *t*-tests for each variable, along with Cohen's *d*, Cronbach's alpha, predictability, and expected influence. The analysis revealed significant gender differences across several variables: cognitive reappraisal (*t* = 2.099, *p*=0.036, Cohen's *d* = 0.208), conversational distress (*t* = −2.875, *p*=0.004, Cohen's *d* = −0.285), socialization and friendship distress (*t* = −2.682, *p*=0.008, Cohen's *d* = −0.266), heterosexual interaction distress (*t* = 3.240, *p*=0.001, Cohen's *d* = 0.321), and social anxiety (t = −4.326, *p* < 0.001, Cohen's *d* = −0.429). The correlation analysis involving latent variables and the dimensions of each scale can be found in the Table S1. Notably, significant correlations were observed among most latent variables except for ER with PEG, NEG, and SA, as well as between PEG and SA.

### 3.2. Network Analysis

**Figure 2:**
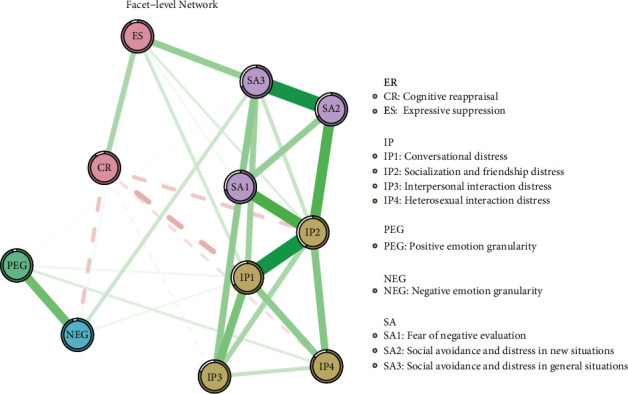
Network structure of facet-level model based on network analysis according to the relationships between emotion regulation, interpersonal relationship, positive emotion granularity, negative emotion granularity and social anxiety and the standardized estimates of node centrality.

Figure 2 illustrates the facet-level network among EG, emotional regulation, interpersonal relationships, and social anxiety. The corresponding node centrality indices for this network are detailed in the Table S3. We identified that 31 of the 55 possible symptom connections (56.4%) were significantly engaged, underscoring robust interconnectivity within the symptomatology. In the Facet network, CR is negatively correlated with IP1, IP2, and IP4, whereas ES shows positive correlations with IP1, IP2, and subdimensions of SA. According to the weight matrix in the Table S2, the edge between SA2 and SA3 exhibits the highest intensity (*r* = 0.382). Notable edge intensities are also observed between IP1 and IP2 (*r* = 0.340), IP2 and SA1 (*r* = 0.255), and IP2 and SA2 (*r* = 0.253). PEG and NEG display strong associations with CR and the dimensions of SA. Apart from the intense correlations within dimensions, the closest relationships are observed between IP2 and SA1, and between IP2 and SA2. In addition, IP1 and IP2 had the highest predictability, at 0.667 and 0.548, respectively, while PEG and NEG showed the lowest predictability, at 0.084 and 0.097, respectively (Table 1).

**Figure 3:**
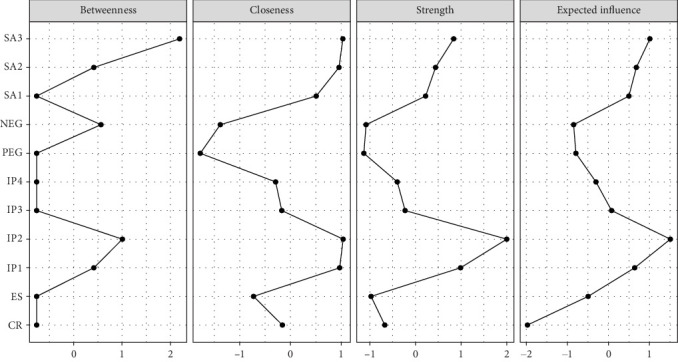
The node centrality indices of the network.

**Figure 4:**
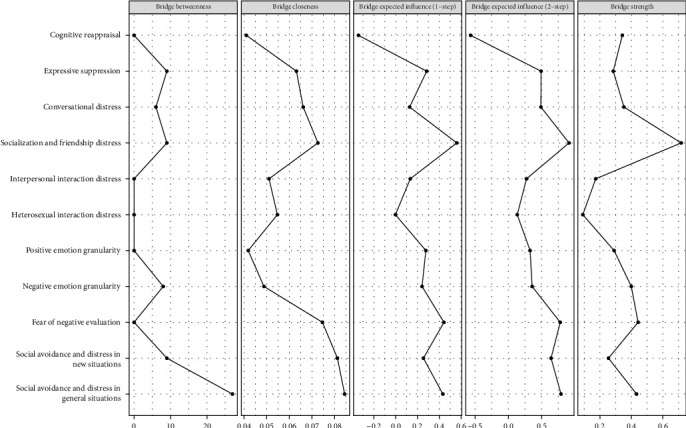
The bridge centrality indices of the network.

Figure 3 and Table S3 reveal that IP2 exhibits the highest strength centrality at 2.002, followed by IP1 and SA3 at 0.989 and 0.838, respectively. Additionally, IP2 also shows the highest closeness centrality, while SA3 ranks highest in betweenness centrality. The expected influences detailed in Table 1 and Figure 3 indicate that IP2 has the greatest expected influence within the network, followed by SA3, and then IP1. A bridge centrality analysis, displayed in Figure 4, shows that SA3 has the highest bridge betweenness and also ranks highest in bridge closeness. Additionally, IP2 displays the highest values in both bridge expected influence and bridge strength.

**Figure 5:**
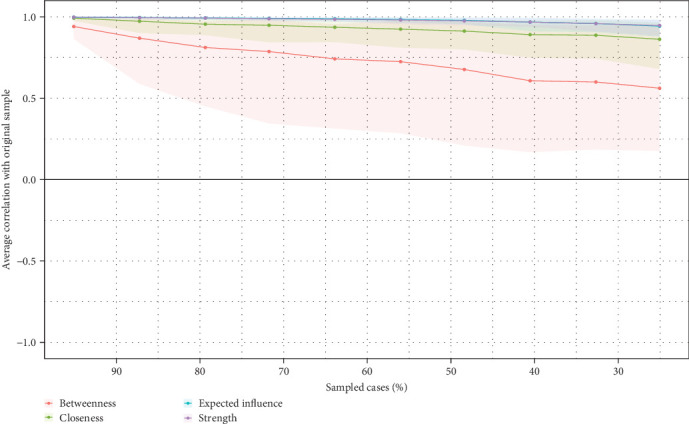
The stability of centrality indices by case dropping subset bootstrap.

Network stability metrics, such as expected influence and strength centrality, have demonstrated high stability with CS coefficients reaching 0.75, as shown in Figure 5. The nonparametric confidence intervals and bootstrapped difference tests further confirm the robustness of these results, as indicated in Figures S1 and S2. Additionally, the edge estimation by bootstrapped difference tests between edge-weights is shown in Figure S3.

The network comparison test revealed a significant difference in the structural distribution of edge weights between genders (*M* = 0.322, *p*=0.005), whereas the global strength and node centrality did not show a significant difference (*S* = 0.943, *p*=0.138).

### 3.3. Bayesian NA

**Figure 6:**
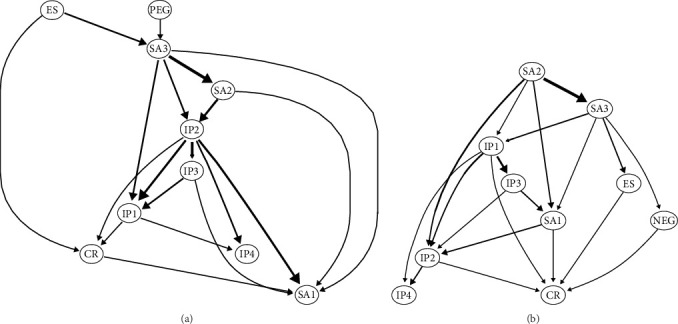
The averaged bootstrapped Bayesian network of positive (A) and negative (B) emotion granularity.

The averaged bootstrapped BN for PEG (Figure 6A) positions the nodes PEG and ES at the apex, directly influencing SA3 and CR. Excluding SA1, nodes SA2 and SA3 within the midsection of the network directly or indirectly impact IP. At the network's terminus, IP4 and SA1 receive the final influences, with IP4 directly influenced by IP1 and IP2, while SA1 is influenced by a combination of nodes including CR, IP2, IP3, SA2, and SA3. In Figure 6A, node SA3 exhibits a high “out-degree,” meaning it has numerous arrows pointing to other nodes, indicating that it may serve as an important antecedent factor influencing other variables. Node CR is targeted by multiple variables (e.g., IP1 and SA3), suggesting that SA3 and IP may jointly affect CR, thereby playing an integrative role within the broader network.

The averaged bootstrapped BN incorporating NEG is presented in Figure 6B. Here, upstream nodes SA2 and SA3 directly impact several downstream nodes, including IP1, SA1, ES, and NEG, with SA2 also influencing IP2. Midstream nodes encapsulate SA and IP constructs, while CR and IP4, as terminal nodes, along with NEG downstream, directly influence CR. In Figure 6B, CR emerges as a more critical variable, connecting to almost all other variables. NEG and ES, on the other hand, play relatively weaker roles within the network, with NEG being connected to other variables through thinner edges.

The regression coefficients for each node within the bootstrapped averaged BN can be found in the Table S5. The regression coefficients for both positive and negative emotion granularity show consistency within the BN, indicating that ES, positively influenced by SA3, predicts CR positively. SA1 is positively predicted by IP2, IP3, and SA3. Additionally, SA2 is positively influenced by IP2, and SA3 by IP1 and SA2.

## 4. Discussions

This study pioneers the use of a BN approach to investigate potential causal relationships among EG, emotion regulation, interpersonal relationships, and social anxiety. Initially, a facet-level undirected network was constructed to examine the interrelationships among these variables. Subsequently, two averaged bootstrapped BNs—one focusing on PEG and the other on NEG—were developed to assess the consistency of their effects on emotion regulation, social anxiety, and interpersonal relationships.

Within the entire network, the connection between the interpersonal relationship nodes IP1 and IP2 is the strongest. IP1 represents an individual's ability to listen attentively to others' opinions and views during conversations and to fully express their feelings. IP2 pertains to an individual's psychological state in social situations or when interacting with others [26]. It is evident that, if an individual can effectively manage their mindset in social situations, they will be more adept at conversations. Thus, the strong correlation between these two nodes is highly plausible. There is a notable association between social anxiety and interpersonal relationships, as evidenced by the strong correlation between node IP2 and node SA2. IP2 and SA2 share many overlapping psychological states. Additionally, high levels of SA2 may lead individuals to avoid social interactions or behave unnaturally, eliciting negative reactions from others and exacerbating interpersonal issues reflected in IP2 [63]. Conversely, difficulties in interpersonal relationships (IP2) can also influence social avoidance and distress (SA2).

The connection between node SA2 and node SA3 represents the second strongest correlation within the entire network. SA2 reflects social avoidance and distress in new social environments or with unfamiliar peers, while SA3 pertains to broader social discomfort maladjustment, and inhibition [64]. Understandably, individuals who score high on SA2 may also score relatively high on SA3, as discomfort experienced in specific environments or with specific individuals can often generalize to broader social contexts. In both PEG and NEG BNs, node SA2 and node SA3 directly impact node IP2 and indirectly influence nodes IP1, IP3, and IP4. Social anxiety and interpersonal relationships are inherently interconnected. SA2 and SA3 represent the withdrawal and anxiety that individuals experience in social environments and towards social partners [64], while IP2 denotes issues of interpersonal distress. According to our study, SA may affect the level of IP, consistent with findings from a study conducted with college students [63].

One of the most significant findings of this study is the highly positive interconnectivity between node PEG and node NEG, addressing a gap in previous research. EG itself represents the ability to differentiate similar emotions, thus testing an individual's emotional awareness and cognitive processing capacity, whether it pertains to positive or negative emotions. Therefore, the consistency between PEG and NEG is evident. Additionally, positive associations were observed between node PEG and node IP4, as well as between node NEG and node SA3.

In the PEG BN (Figure 6A), nodes PEG and ES are positioned at the top, exerting influence on downstream variables such as SA3 and IP. Node CR was influenced by nodes IP and ES, with node ES having a positive regression on node CR. This indicates that PEG and ES jointly affect SA3, meaning that individuals with lower PEG tend to exhibit more social avoidance and anxiety. Additionally, the frequent use of ES strategies also exacerbates SA3 symptoms. As a result of the excessive use of ES and the continuous emergence of social and interpersonal problems, individuals may increase their use of CR strategies. Previous research has indicated that regardless of PEG ability, individuals with high social anxiety (HSA) tend to use ES strategies, Moreover, those with poor EG among HSA individuals use CR strategies less frequently, indicating inflexible emotion regulation [40].

However, the difference is that in the NEG BN (Figure 6B), nodes NEG and ES are situated midstream and indirectly influence nodes IP2 and IP4, while node SA3 directly impacts both, which is the opposite of the results in the PEG BN. Additionally, SA2 is positioned at the top of the network, directly influencing SA3. This indicates that individuals with severe social avoidance (whether in general or specific situations) are more likely to use ES to alleviate their anxiety, consistent with previous research [39]. Due to reduced social interactions, individuals with social anxiety tend to have lower negative emotion granularity, making it increasingly difficult for them to differentiate between negative emotions, thus exacerbating their social anxiety symptoms.

Additionally, besides ES, CR is also affected by NEG. This suggests that individuals with lower NEG are more inclined to use CR strategies. As the effectiveness of ES diminishes with repeated use, individuals may increase their use of CR strategies accordingly. Studies have shown that the level of EG influences the relationship between emotions and intrinsic motivation for both positive and negative emotions [65]. Individuals with lower PEG exhibit a stronger connection between specific positive emotions and intrinsic motivation, whereas those with higher NEG display a weaker connection between specific negative emotions and intrinsic motivation [65]. This also explains why NEG does not directly influence individuals' behavioral tendencies.

This study also confirmed that females scored higher on SA and IP, which is consistent with previous research showing that adolescent girls are more concerned about how others judge their appearance and behavior and experience more interpersonal difficulties [66]. Additionally, females' lower CR scores may be related to their higher SA scores, and previous studies have shown that higher levels of social anxiety are associated with lower cognitive reassessment effects [39]. Furthermore, the ICCs indicated that girls are better at categorizing similar emotions than boys. However, girls displayed lower emotion regulation efficacy, potentially related to the scale's suitability for adolescents.

This study has several limitations. First, the sample size is restricted due to constraints inherent in the standardized experimental design. Future research should aim to expand the sample size to enhance result accuracy. While the findings of this study may be applicable to middle school students in China, caution should be exercised when attempting to generalize these findings to other age groups or regions. Second, the young age of participants in this study might introduce biases in their comprehension of the emotion granularity task. To ensure robust conclusions in future studies with adolescents, it is advisable to employ more precise measurement methods or multiple concurrent techniques, such as ES and EMA. Additionally, to reduce self-report biases, qualitative research methods such as naturalistic observation or individual interviews could be utilized. Lastly, given that the current study is cross-sectional, its findings are inherently limited. For a more comprehensive understanding of symptom changes over time, future research should consider longitudinal studies or interventions. Employing NA in such studies could provide more accurate interpretations of longitudinal data and may also reveal unexpected results. Despite these limitations, focusing on adolescents is valuable as it expands research into a relatively underexplored demographic and has the potential to improve their emotional skills and overall well-being. Future initiatives should design EG training suitable for adolescents, which can help enhance their interpersonal relationships and mental health. Research has shown that mindfulness interventions can enhance EG, with studies finding that mindfulness is linked to greater emotion differentiation and fewer emotional difficulties [67, 68]. Additionally, other studies have focused on increasing EG among adolescents by teaching them emotion-related vocabulary, also known as vocabulary building, a method found to be particularly effective for this age group[13].

## 5. Conclusion

This study employed NA to construct both undirected and directed network models, aiming to elucidate potential causal relationships involving PEG and NEG concerning emotion regulation, social anxiety, and interpersonal relationships. The findings reveal that PEG directly influences social anxiety symptoms and interpersonal issues and indirectly affects CR. Conversely, NEG is primarily influenced by social anxiety symptoms and directly affects CR strategies. Additionally, the use of ES is linked to an increase in CR strategies, with NEG also contributing to this effect. Consequently, interventions targeting EG—whether positive or negative—can be effective in improving interpersonal relationships and reducing social anxiety among adolescents.

## Figures and Tables

**Table 1:** The descriptive statistics results of each variable.

Variables	Males (*n* = 204)	Females (*n* = 203)	*t*	*P*	Cohen's *d*	Cronbach's alpha	Predictability^a^	Expected influence^b^
Emotion regulation	57.250 ± 12.248	55.803 ± 11.883	1.210	0.227	0.120	0.771	—	—
Cognitive reappraisal	31.461 ± 8.502	29.650 ± 8.897	2.099	0.036	0.208	—	0.180	−1.973
Expressive suppression	25.789 ± 7.602	26.153 ± 7.424	−0.488	0.626	−0.048	—	0.141	−0.495
Interpersonal relationship	8.152 ± 5.335	8.897 ± 5.398	−1.399	0.162	−0.139	0.852	—	—
Conversational distress	2.029 ± 1.808	2.557 ± 1.891	−2.875	0.004	−0.285	—	0.548	0.639
Socialization and friendship distress	2.740 ± 1.827	3.246 ± 1.977	−2.682	0.008	−0.266	—	0.668	1.509
Interpersonal interaction distress	1.539 ± 1.457	1.749 ± 1.368	−1.496	0.136	−0.148	—	0.351	0.077
Heterosexual interaction distress	1.843 ± 1.617	1.345 ± 1.482	3.240	0.001	0.321	—	0.266	−0.304
Positive emotion granularity	0.278 ± 0.213	0.260 ± 0.198	0.896	0.371	0.089	—	0.084	−0.793
Negative emotion granularity	0.163 ± 0.157	0.153 ± 0.120	0.672	0.502	0.067	—	0.096	−0.848
Social anxiety	25.789 ± 9.414	29.941 ± 9.941	−4.326	<0.001	−0.429	0.902	—	—
Fear of negative evaluation	9.990 ± 4.417	11.488 ± 4.374	−3.437	<0.001	−0.341	—	0.471	0.499
Social avoidance and distress in new situations	8.417 ± 3.702	10.374 ± 4.184	−5.000	<0.001	−0.496	—	0.541	0.680
Social avoidance and distress in general situations	7.382 ± 3.114	8.079 ± 3.209	−2.222	0.027	−0.220	—	0.517	1.010

^a,b^The values of predictability and expected influence were generated from the facet-level network.

## Data Availability

The data that support the findings of this study are available from the corresponding author upon reasonable request.

## Nomenclature

NA: Network analysis
GGM: Graphical Gaussian model
ICC: Intraclass correlation coefficient
PED task: Photo emotion differentiation task
EG: Emotional granularity
PEG: Positive emotion granularity
NEG: Negative emotion granularity
ER: Emotion regulation
CR: Cognitive reappraisal
ES: Expressive suppression
IP: Interpersonal relationship
IP1: Conversational distress
IP2: Socialization and friendship distress
IP3: Interpersonal interaction distress
IP4: Heterosexual interaction distress
SA: Social anxiety
SA1: Fear of negative evaluation
SA2: Social avoidance and distress in new situations
SA3: Social avoidance and distress in general situations.

## Supporting Information

Supporting Information Table S1: correlation analysis of the all variables. Table S2: edge weight matrix of the facet-level network. Table S3: the node centrality indices of the facet-level network. Table S4: the items of ERS, IRCDS, and SAS-A. Table S5: the regression coefficients of each dimension node in bootstrapped averaged BN of positive emotion granularity or negative emotion granularity. Figure S1: the regression coefficients of each dimension node in bootstrapped averaged BN of PEG and NEG. Figure S2: bootstrapped confidence intervals of edge weights (Smaller CIs indicate that the estimation of edges is more accurate). Figure S3: edge estimation by bootstrapped difference tests between edge-weights (Gray boxes indicate nodes or edges that do not differ significantly from one another and black boxes represent nodes or edges that do differ significantly from one another).
