# Supplementary material for: Emotional Granularity and Cognitive Reappraisal Affect Social Anxiety and Interpersonal Relationships in Adolescents: A Bayesian Network Analysis
Source: Depress Anxiety. 2025 Feb 27;2025:8658973. doi: 10.1155/da/8658973 (PMC11987076; doi:10.1155/da/8658973)

**Supplementary materials**

Table S1.

|  | 1 | 2 | 3 | 4 | 5 | 6 | 7 | 8 | 9 | 10 | 11 | 12 | 13 |
| --- | --- | --- | --- | --- | --- | --- | --- | --- | --- | --- | --- | --- | --- |
| 1. ER |  |  |  |  |  |  |  |  |  |  |  |  |  |
| 2. CR | 0.786*** |  |  |  |  |  |  |  |  |  |  |  |  |
| 3. ES | 0.694*** | 0.100* |  |  |  |  |  |  |  |  |  |  |  |
| 4. IP | -0.098* | -0.328*** | 0.224*** |  |  |  |  |  |  |  |  |  |  |
| 5. IP1 | -0.074 | -0.301*** | 0.231*** | 0.855*** |  |  |  |  |  |  |  |  |  |
| 6. IP2 | -0.068 | -0.305*** | 0.245*** | 0.861*** | 0.681*** |  |  |  |  |  |  |  |  |
| 7. IP3 | -0.094 | -0.195*** | 0.076 | 0.718*** | 0.508*** | 0.493*** |  |  |  |  |  |  |  |
| 8. IP4 | -0.079 | -0.216*** | 0.124* | 0.708*** | 0.448*** | 0.471*** | 0.350*** |  |  |  |  |  |  |
| 9. PEG | -0.091 | -0.124* | -0.003 | 0.100* | 0.100* | 0.068 | 0.021 | 0.123* |  |  |  |  |  |
| 10. NEG | -0.052 | -0.139** | 0.078 | 0.102* | 0.104* | 0.086 | 0.036 | 0.089 | 0.270*** |  |  |  |  |
| 11. SA | 0.087 | -0.169*** | 0.289*** | 0.646*** | 0.557*** | 0.708*** | 0.389*** | 0.334*** | 0.081 | 0.137*** |  |  |  |
| 12. SA1 | 0.076 | -0.088 | 0.224*** | 0.567*** | 0.441*** | 0.611*** | 0.433*** | 0.280*** | 0.018 | 0.106* | 0.843*** |  |  |
| 13. SA2 | 0.023 | -0.170*** | 0.236*** | 0.532*** | 0.471*** | 0.621*** | 0.261*** | 0.266*** | 0.076 | 0.098* | 0.868*** | 0.550*** |  |
| 14. SA3 | 0.043 | -0.184*** | 0.285*** | 0.536*** | 0.512*** | 0.553*** | 0.270*** | 0.308*** | 0.132** | 0.154** | 0.821*** | 0.517*** | 0.650*** |

Table S2.

| **Variable** | CR | ES | IP1 | IP2 | IP3 | IP4 | PEG | NEG | SA1 | SA2 | SA3 |
| --- | --- | --- | --- | --- | --- | --- | --- | --- | --- | --- | --- |
| CR | 0.000 | 0.093 | -0.101 | -0.095 | -0.010 | -0.058 | -0.034 | -0.053 | 0.000 | 0.000 | 0.000 |
| ES | 0.093 | 0.000 | 0.048 | 0.041 | 0.000 | 0.000 | 0.000 | 0.000 | 0.031 | 0.017 | 0.114 |
| IP1 | -0.101 | 0.048 | 0.000 | 0.340 | 0.206 | 0.147 | 0.000 | 0.000 | 0.000 | 0.000 | 0.166 |
| IP2 | -0.095 | 0.041 | 0.340 | 0.000 | 0.123 | 0.185 | 0.000 | 0.000 | 0.255 | 0.253 | 0.057 |
| IP3 | -0.010 | 0.000 | 0.206 | 0.123 | 0.000 | 0.111 | 0.000 | 0.000 | 0.145 | 0.000 | 0.000 |
| IP4 | -0.058 | 0.000 | 0.147 | 0.185 | 0.111 | 0.000 | 0.028 | 0.000 | 0.000 | 0.000 | 0.009 |
| PEG | -0.034 | 0.000 | 0.000 | 0.000 | 0.000 | 0.028 | 0.000 | 0.199 | 0.000 | 0.000 | 0.029 |
| NEG | -0.053 | 0.000 | 0.000 | 0.000 | 0.000 | 0.000 | 0.199 | 0.000 | 0.000 | 0.000 | 0.054 |
| SA1 | 0.000 | 0.031 | 0.000 | 0.255 | 0.145 | 0.000 | 0.000 | 0.000 | 0.000 | 0.170 | 0.146 |
| SA2 | 0.000 | 0.017 | 0.000 | 0.253 | 0.000 | 0.000 | 0.000 | 0.000 | 0.170 | 0.000 | 0.382 |
| SA3 | 0.000 | 0.114 | 0.166 | 0.057 | 0.000 | 0.009 | 0.029 | 0.054 | 0.146 | 0.382 | 0.000 |

Table S3.

| **Variables** | **Betweenness** | **Closeness** | **Strength** | **Expected influence** |
| --- | --- | --- | --- | --- |
| CR | -0.766 | -0.159 | -0.673 | -1.973 |
| ES | -0.766 | -0.727 | -0.974 | -0.495 |
| IP1 | 0.417 | 0.970 | 0.989 | 0.639 |
| IP2 | 1.008 | 1.039 | 2.002 | 1.509 |
| IP3 | -0.766 | -0.171 | -0.228 | 0.077 |
| IP4 | -0.766 | -0.292 | -0.399 | -0.304 |
| PEG | -0.766 | -1.777 | -1.132 | -0.793 |
| NEG | 0.564 | -1.382 | -1.084 | -0.848 |
| SA1 | -0.766 | 0.508 | 0.222 | 0.499 |
| SA2 | 0.417 | 0.957 | 0.440 | 0.680 |
| SA3 | 2.190 | 1.033 | 0.838 | 1.010 |

Table S4.

| **Scale** | **Items** |
| --- | --- |
| ERS | Reappraisal factor  When faced with a situation that makes me angry, I alter my perspective on the issue to alleviate my anger. |
|  | I modify my way of thinking to reduce my aversion to someone or something. |
|  | I change the way I interpret the situation to manage my emotions. |
|  | When faced with a situation that makes me sad, I think about it from a different perspective to lessen my sadness. |
|  | I attempt to change my perception of the surrounding environment to make myself happier.  When faced with a situation that scares me, I change my view of the situation to reduce my fear.  I change my way of thinking to regulate my emotions.  Suppression factor |
|  | When I feel happy, I make an effort not to express it outwardly. |
|  | When I feel sad, I suppress this emotion to prevent others from knowing my true feelings. |
|  | I do not exhibit my emotions. |
| IRCDS | When I feel fearful, I do not let my emotions show. |
|  | When I am angry, others cannot tell from my appearance that I am angry inside.  I control my emotions by not expressing them. |
|  | When I dislike someone or something, I suppress this feeling to avoid showing it. |
|  |  |
|  | Interpersonal Conversation |
|  | Difficulties expressing my own troubles verbally. |
|  | Feeling difficult to continue talking continuously. |
|  | Often feeling lonely or isolated when with a group of friends. |
|  | Feeling uncomfortable when a stranger confides their life experiences to me. |
|  | Often avoiding expressing my own feelings. |
|  | Unable to focus on listening. |
|  | Unable to objectively consider various opinions and viewpoints.  Socialization and Friendship  Feeling awkward when meeting people for the first time. |
|  | Feeling awkward in social situations. |
|  | Feeling extremely vulnerable. |
|  | Concerned about others' opinions of me. |
|  | Lacking confidence in my appearance. |
|  | Having no one to confide my troubles in. |
|  | Feeling secretly hurt due to the suffering of others. |
|  | Interpersonal Interaction |
|  | Excessive shyness and timidity towards others. |
|  | Occasionally hurting others. |
|  | Unable to get along well with others. |
|  | Always trying hard to make others appreciate me. |
|  | Avoiding discussions about others or being discussed by them. |
|  | Feeling resentful towards exclusion and coldness from others. |
|  | Often talking nonsense or behaving foolishly. |
|  | Heterosexual Interaction |
|  | Minimal interaction with the opposite sex. |
|  | Feeling awkward when interacting with the opposite sex. |
|  | Not knowing how to appropriately interact with the opposite sex. |
|  | Secretly brooding over sex. |
|  | Looking down on the opposite sex. |
|  | Being looked down upon by the opposite sex. |
|  | Unsure how to improve relationships with the opposite sex. |
|  |  |
| SAS-A | Fear of Negative Evaluation  I worry about what others think of me. |
|  | I am afraid that others will not like me. |
|  | I worry about what others say about me. |
|  | I worry that others don’t like me. |
|  | Social Avoidance and Distress-New  I feel shy around people I don’t know. |
|  | I get nervous when I talk to peers I don’t know very well. |
|  | I get nervous when I meet new people. |
|  | I feel nervous when I’m around certain people.  Social Avoidance and Distress-General  I am quiet when I’m with a group of people.  I’m afraid to invite others to do things with me because they might say no.  I feel shy even with peers I know very well.  It’s hard for me to ask others to do things with me. |

Table S5.

| **nodes** | **Coefficients (PEG/NEG)** |
| --- | --- |
| CR.(Intercept) | -0.0005340537 |
| CR.ES | 0.0981687449 |
| ES.(Intercept) | -0.0057822355 |
| ES.SA3 | 0.3149012089 |
| IP1.(Intercept) | 0.0160506855 |
| IP1.IP2 | 0.6629771574 |
| IP3.(Intercept) | 0.0132449027 |
| IP3.IP1 | 0.4883370739 |
| IP4.(Intercept) | -0.0288037400 |
| IP4.IP1 | 0.2349954792 |
| IP4.IP2 | 0.2887057757 |
| SA1.(Intercept) | -0.0100090473 |
| SA1.IP2 | 0.3926551967 |
| SA1.IP3 | 0.1678727016 |
| SA1.SA3 | 0.2635188784 |
| SA2.(Intercept) | 0.0051770614 |
| SA2.IP2 | 0.6190010685 |
| SA3.(Intercept) | 0.0097138181 |
| SA3.IP1 | 0.2661515704 |
| SA3.SA2 | 0.5001826953 |

Figure S1.


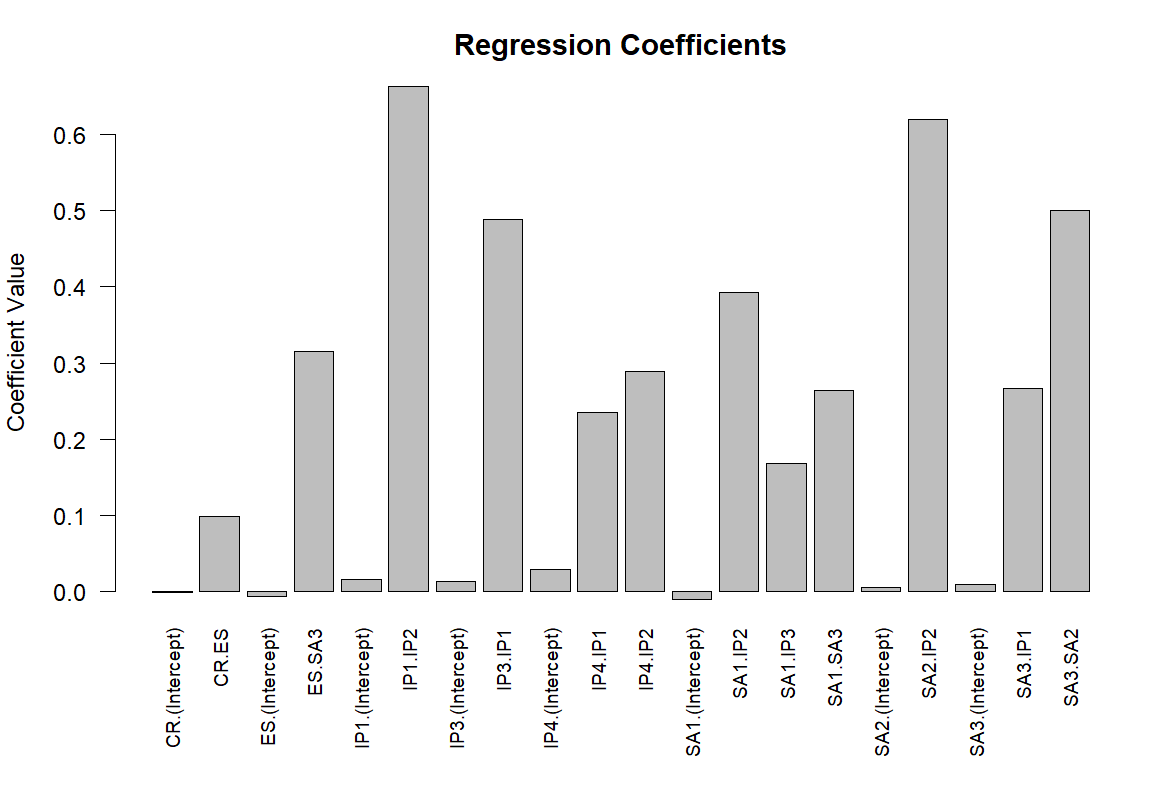


Figure S2.


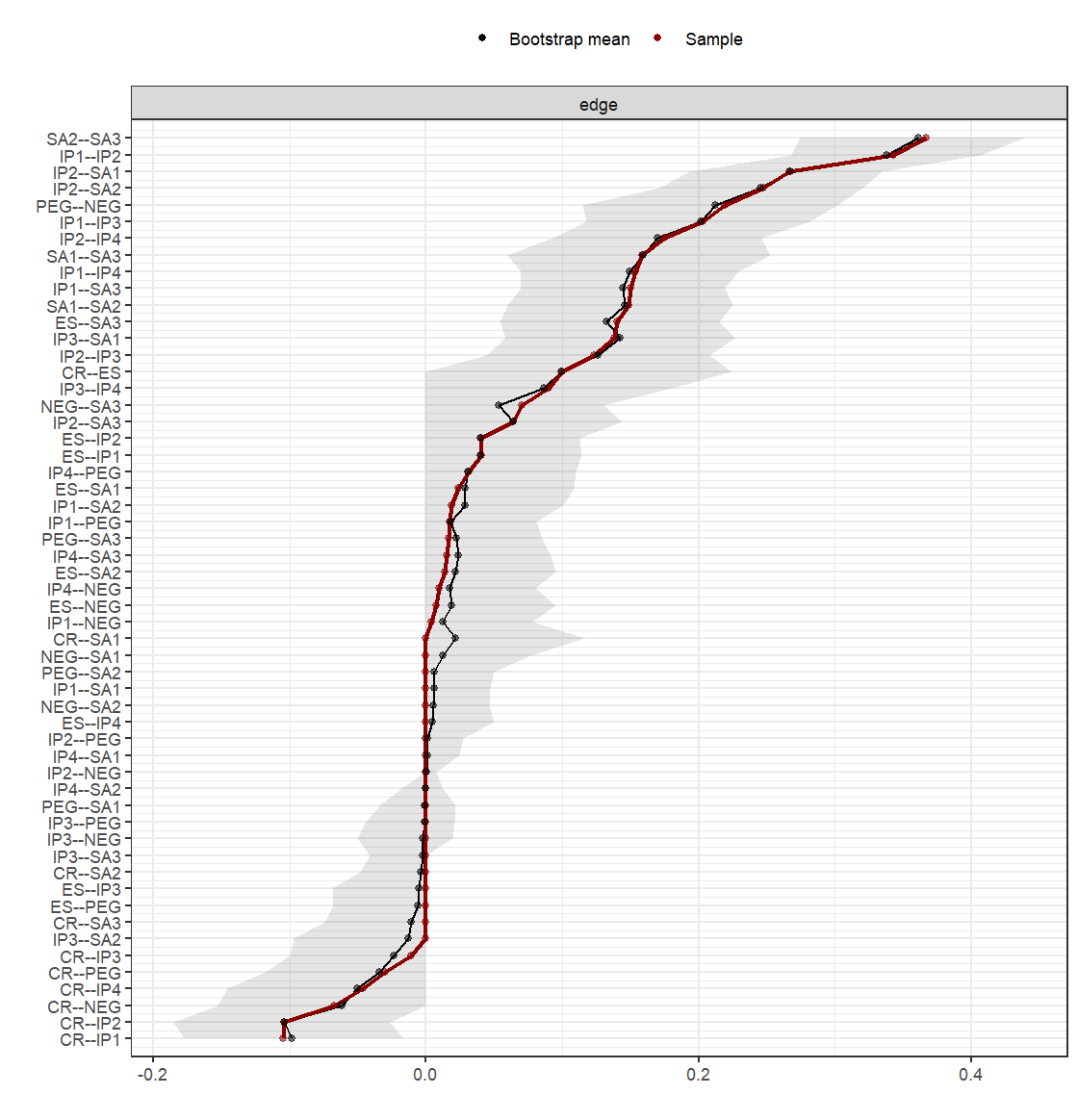


Figure S3.


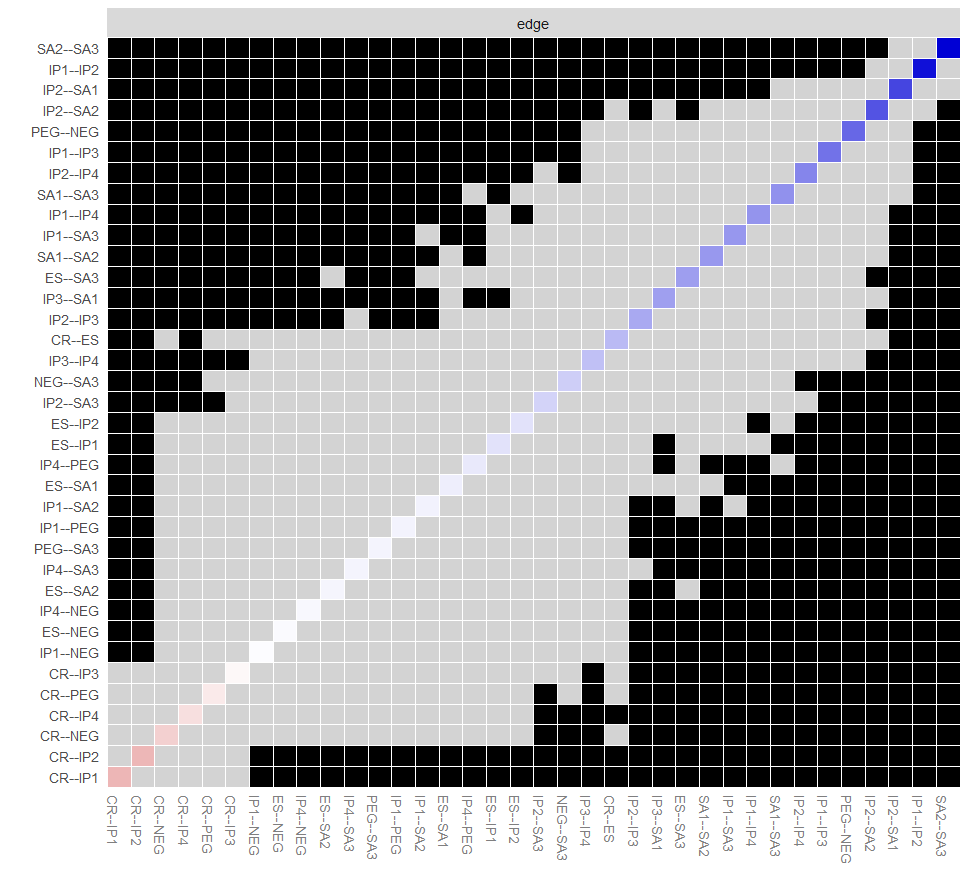

Supplement: Supporting Information — Table S1: correlation analysis of the all variables. Table S2: edge weight matrix of the facet-level network. Table S3: the node centrality indices of the facet-level network. Table S4: the items of ERS, IRCDS, and SAS-A. Table S5: the regression coefficients of each dimension node in bootstrapped averaged BN of positive emotion granularity or negative emotion granularity. Figure S1: the regression coefficients of each dimension node in bootstrapped averaged BN of PEG and NEG. Figure S2: bootstrapped confidence intervals of edge weights (Smaller CIs indicate that the estimation of edges is more accurate). Figure S3: edge estimation by bootstrapped difference tests between edge-weights (Gray boxes indicate nodes or edges that do not differ significantly from one another and black boxes represent nodes or edges that do differ significantly from one another). [file 8658973.f1.docx]
